# From perception to action: phase-locked gamma oscillations correlate with reaction times in a speeded response task

**DOI:** 10.1186/1471-2202-8-27

**Published:** 2007-04-17

**Authors:** Ingo Fründ, Niko A Busch, Jeanette Schadow, Ursula Körner, Christoph S Herrmann

**Affiliations:** 1 Institute of Psychology, Department of Biological Psychology, Otto-von-Guericke University, Magdeburg, Germany; 2 Honda Research Institue Europe, Offenbach, Germany

## Abstract

**Background:**

Phase-locked gamma oscillations have so far mainly been described in relation to perceptual processes such as sensation, attention or memory matching. Due to its very short latency (≈90 ms) such oscillations are a plausible candidate for very rapid integration of sensory and motor processes.

**Results:**

We measured EEG in 13 healthy participants in a speeded reaction task. Participants had to press a button as fast as possible whenever a visual stimulus was presented. The stimulus was always identical and did not have to be discriminated from other possible stimuli. In trials in which the participants showed a fast response, a slow negative potential over central electrodes starting approximately 800 ms before the response and highly phase-locked gamma oscillations over central and posterior electrodes between 90 and 140 ms after the stimulus were observed. In trials in which the participants showed a slow response, no slow negative potential was observed and phase-locked gamma oscillations were significantly reduced. Furthermore, for slow response trials the phase-locked gamma oscillations were significantly delayed with respect to fast response trials.

**Conclusion:**

These results indicate the relevance of phase-locked gamma oscillations for very fast (not necessarily detailed) integration processes.

## Background

Response times to visual stimuli can be extremely fast in some cases. However, there is also considerable intra-individual variability in response times across trials, even under identical experimental conditions. Which neural processes can account for these behavioral differences? Why do we manage to be fast on some trials but not on others?

Experimental findings suggest that processing of visual information may be extremly rapid, leaving an upper limit of 10–20 ms for information transfer between two neurons at successive levels of the visual processing hierarchy [1]. This implies that processing must be achieved using the very first spikes of a neuronal stimulus response based on an ensemble code rather than a rate code [2]. Such effective and fast transfer demands synchronous arrival of several spikes at the same target neuron, propagated from different source neurons that were activated by the respective stimulus. However, the membrane potential of neurons is randomly fluctuating around its resting potential. Therefore, without a synchronization of those fluctuations even a coherent wave of input spikes to a certain processing stage will result in an increasingly scattered spike distribution at the input to the next processing stage. In this case reliable processing of a stimulus can only be achieved based on a time consuming rate code. Rodemann and Körner [3] demonstrated in a neural network simulation that stimulus-locked, evoked gamma band responses (eGBRs) can be the expression of a phase reset of ongoing neuronal activity after a visual stimulus, which results in strong synchronization of spiking activity in the stimulated neuronal population. In this case processing of the stimulus can be based on instantaneous evaluation of an ensemble code, which results in much faster responses.

So far eGBRs have been studied in perceptual tasks in both auditory [4,5] and visual modalities [6,7]. In these experiments it could be shown that although eGBRs are highly dependent on physical parameters of the stimulation [6,8], they are significantly modulated by top down factors like attention [5,9] or memory [10]. Although the early time window in which they occur and the simulation performed by Rodemann and Körner [3] render eGBRs a possible mechanism for fast response initiation, studies that link eGBRs and response times are rare. There is only indirect evidence regarding this topic. In studies investigating eGBRs in schizophrenic patients, decreased phase-locking is accompanied by prolongated reaction times in patients compared to healthy controls [11]. Furthermore, studies that investigated stimulus induced amplitude modulations in the gamma range found relations between such amplitude modulations and reaction time [12,13]. Such later amplitude modulations of oscillations in the gamma range, so called induced gamma oscillations [14] have been associated with a wide range of cognitive processes [15,16] and learning [17,18].

In the current study, we directly investigated the idea that phase-locked, evoked GBRs are relevant for speeded responses. Participants were asked to respond as fast as possible to large black squares presented on a white screen, while their electroencephalogram (EEG) was recorded. Trials in which the participants were able to respond fast were analyzed separately from trials with slower reactions. We hypothesized that eGBRs would be enhanced in those trials, in which the participants were able to respond fast, compared to trials, in which participants responded slower. We further explored whether these effects could be explained by amplitude modulations in the single trials or whether they were due to an increase in phase-locking to the onset of the stimulus.

## Results

### Response times

**Figure 1:**
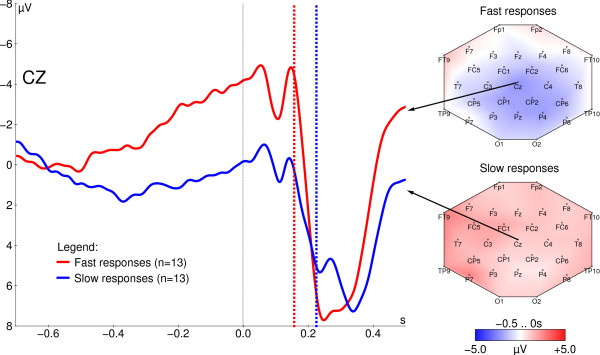
Averaged event related potentials for fast and slow responses (left) and topographic maps of the average activity in the time window -0.5 to 0 s. The stimulus was presented at 0 s. Dotted lines indicate mean response times of fast response trials (red) and slow response trials (blue). Note that the negative potential starting approximately 700 ms before stimulus onset for fast response trials is virtually absent for slow response trials.

**Figure 2:**
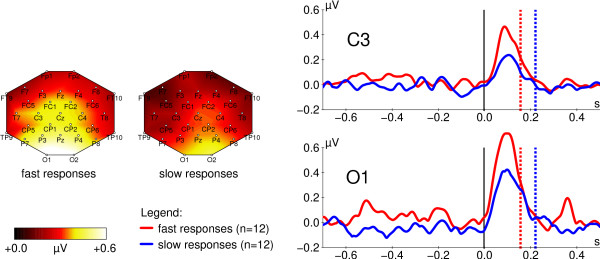
Evoked gamma band responses for fast and slow motor responses (right) and topographic maps of the evoked gamma responses in the time range 60 to 130 ms (left) averaged across all participants. The vertical black lines indicate stimulus onset, dotted lines indicate mean response times of fast response trials (red) and slow response trials (blue). Note the marked increase of the response for fast response trials.

**Figure 3:**
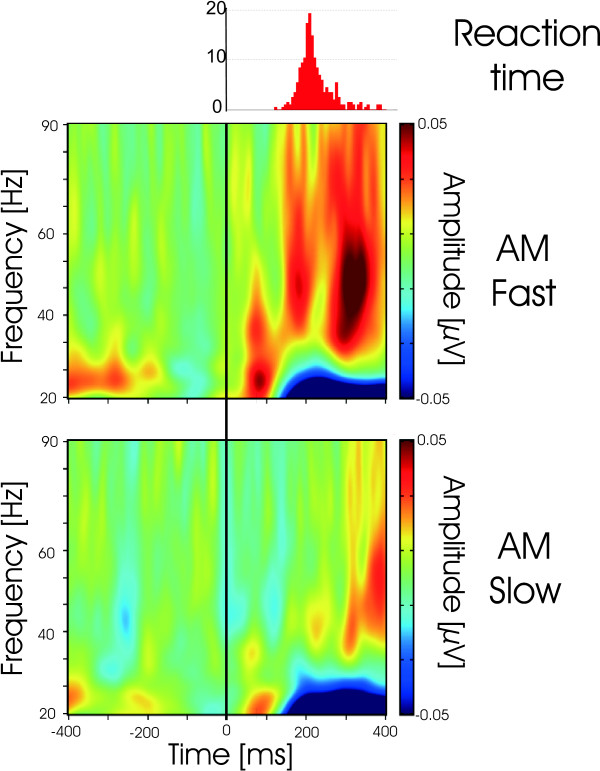
AM patterns for fast and slow motor responses and reaction time histogram. Top: Reaction time histogram of all trials from all participants. Time axis is like below. Middle: Time frequency representation of AM for fast response trials. Bottom: Time frequency representation of AM in slow response trials. Data from the posterior ROI have been averaged to obtain the time frequency representations. Stimulus onset is at 0 ms. Note that the response time histogram peaks considerably earlier than the gamma activity.

Median response times ranged between 170 and 236 ms (mean = 204 ms, standard deviation = 16 ms). Mean response times for fast trials (faster, than the median response time, red) and slow trials (slower, than the median response time, blue) are indicated in Figures 1 and 2 as vertical dotted lines. A histogram of all response times can be found at the top of Figure 3.

### Event related potentials

A slowly increasing negative potential could be observed preceding the stimulus. In order to test whether this negativity is influenced by the degree of expectancy of the next stimulus, we used three different randomizations of the inter stimulus interval (ISI): a uniform distribution, for which all possible ISIs had the same probability, a gaussian distribution with a clear peak for medium latency ISIs, an exponential distribution, for which very long ISIs are possible, but most ISIs are relatively short. These three ISI distributions have been shown to influence the slow stimulus preceding potentials [19]. In the current data, however, the negative potential did not differ between different randomizations of the inter stimulus interval (*F*(2, 12) = 0.51) and was most pronounced over central electrodes. Separating the trials into fast and slow responses revealed that this slowly increasing negative potential was large for fast response trials, but nearly absent for slow response trials (Fig. 1). Comparing the mean amplitude in the last 500 ms preceding stimulus onset in a central region of interest (ROI) yielded a highly significant difference (*t*(12) = -6.233, *p *< 10^-4^).

In addition also a difference in P1-N1 peak to peak amplitude is visible in Figure 1. However, this difference was only visible in a small subset of three participants. In some participants no P1-N1 complex could be found or the effect was even reversed. A statistical analysis of those participants for which a P1-N1 pattern could be observed did not yield a significant difference between fast and slow responses (*t*(10) = -1.71).

### Gamma band responses

Gamma band responses (GBRs) were quantified by three different parameters. Evoked GBRs (eGBRs) were obtained from the wavelet transform of the averaged event-related potential, gamma band amplitude modulation (AM) was computed as the average amplitude of the single trial wavelet transforms and gamma band phase-locking (PL) was quantified as phase-average (mean resultant length, [20,21]) of the single trial wavelet transforms.

A clear eGBR could be observed over posterior and central areas. This response peaked between 50 and 160 ms at frequencies between 30 and 50 Hz (see Table 1). Evoked GBRs were generally more pronounced over the posterior ROI than over the central ROI (*F*(1, 9) = 28.91, *p *< 0.001). No significant differences for different ISI randomizations were found (*F*(2, 9) = 0.29). Separating the trials into fast and slow behavioral responses yielded a clear difference in the eGBR (see Fig. 2): In trials with fast behavioral responses, eGBRs were larger in amplitude (*F*(1, 9) = 12.36, *p *< 0.01) and earlier in latency (*F*(1, 9) = 5.54, *p *< 0.05) than in trials with slow behavioral responses. No significant differences were observed in the baseline level of gamma activity (*t*(11) = 0.36).

**Figure 4:**
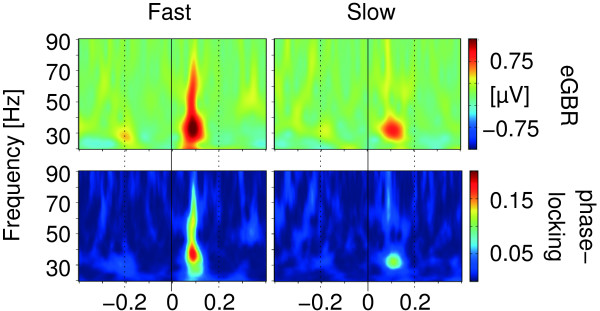
Time frequency representations of eGBR (top) and phase-locking (bottom) for fast responses (left) and slow responses (right) of a single representative participant. Both measures show a considerable enhancement for fast responses.

We analyzed phase-locking (PL) and amplitude modulation (AM) patterns in the same time window to investigate whether the effects of eGBR were due to an increased phase-locking to the stimulus or due to amplitude modulations in the single trials. PL was significantly enhanced for the fast response trials compared to the slow response trials (*F*(1, 10) = 9.30, *p *< 0.05, Fig. 4). PL was also more pronounced over the posterior ROI than over the central ROI (*F*(1, 10) = 11.63, *p *< 0.01). AM was more pronounced over the posterior ROI (*F*(1, 9) = 30.77, *p *< 0.001), too. However, AM between 50 and 160 ms did not depend on the response speed (*F*(1, 9) = 0.83). In a later time window between 180 and 400 ms AM was significantly modulated by reponse speed (*F*(1, 9) = 8.51, *p *< 0.05, Fig. 3). Note however, that this effect was found in most cases after the participants had already pressed the button.

### Relation between stimulus preceding ERP and gamma band response

**Figure 5:**
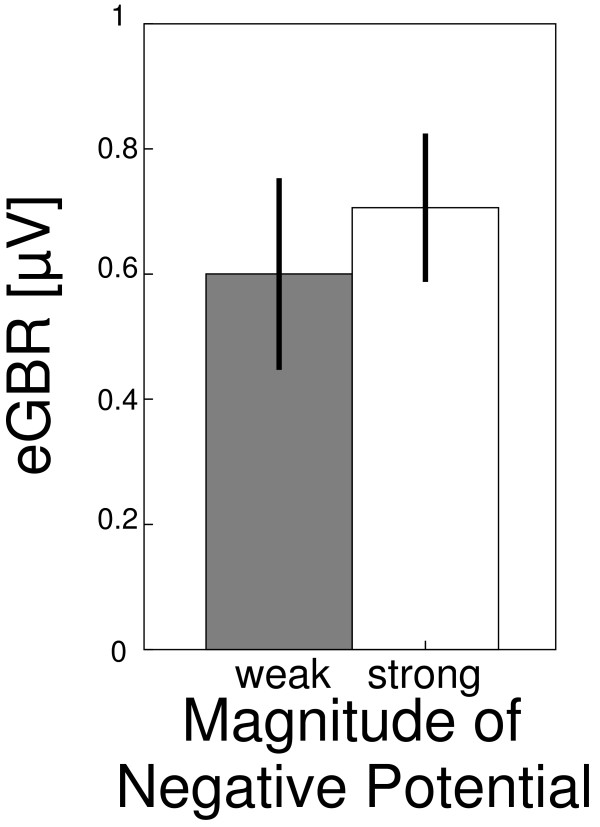
Strength of the eGBR in trials with weak and strong negative potential preceding the stimulus. Subaverages with weak negative potential are marked in grey, subaverages with strong negative potential are marked in white. Error bars indicate standard error of mean. Note the large error bars, that result from the fact that less than half of the participants responded with an enhanced evoked gamma peak in strong negativity trials, while this effect was even reversed in some participants.

In order to disentangle the relation between the slow negative potential and the eGBR, we split the trials into two groups of trials with either a pronounced prestimulus negativity (strong negativity trials) or a weak prestimulus negativity (weak negativity trials) and analyzed eGBRs separately in both subsets. No significant eGBR differences were found between trials with strong and weak negativity (*F*(1, 9) = 0.96). As depicted in Figure 5, this was due to large standard deviations between single participants. Inspection of single participant data revealed that out of 11 participants, five demonstrated enhanced eGBRs in strong negativity trials compared to weak negativity trials, while two participants demonstrated decreased eGBRs in strong negativity trials compared to weak negativity trials. For the remaining four participants eGBRs were virtually the same for strong negativity trials compared to weak negativity trials.

## Discussion

In the current study we demonstrated that both ERPs and eGBRs are increased for fast compared to slow responses. We further pointed out that the enhanced eGBRs for fast responses are a result of increased phase-locking to the stimulus, rather than stimulus related amplitude modulations.

We observed a slowly increasing potential that appeared 500 ms before stimulus onset and was terminated by the participant's response. On the one hand, this negativity could reflect a contingent negative variation (CNV, [22]). Such a CNV would be expected to vary between blocks depending on the degree of expectancy, i.e. the distribution of inter stimulus intervals [19]. However, in our paradigm no significant differences were found between the three blocks which differed in the distribution of inter stimulus intervals and thus in the degree of expectancy. On the other hand, the negativity might also reflect a readiness potential [23-25]. However, a readiness potential should normally be observed preceding self paced movements [26], whereas in the current study no such movements were required. We demonstrated that this negative potential differed between fast and slow responses, which is in line with previous studies, that showed that reaction times are short if a pronounced readiness potential can be observed [23]. Deecke et al. [25] have argued that readiness potentials are recordable only before voluntary movements (actions) but not or to a lesser degree before reactions as in case of the reaction to a visual stimulus. However, other authors also described readiness potentials before reactions to a stimulus [24,27,28]. Thus, based on our current data we cannot discriminate between a CNV and a readiness potential that preceds the stimulus. However, both types of slow potentials have been associated with anticipatory motor preparation [26]. 

We could extend the ERP findings by showing a relation between evoked gamma oscillations and reaction time, indicating that fast reactions are associated with highly phase-locked gamma oscillations. In line with the ERP results this effect was observed at central electrodes [23]. However, in the current study we found significant differences between fast and slow response trials also at posterior electrodes. Previous results indicating that eGBRs are highly dependend on physical factors of the stimulus, linked eGBRs to very early visual processing [6]. This extends the findings obtained from ERP analysis by showing that fast and slow response trials differ already in earlier stages of visual processing. The current findings are in line with both findings from the auditory modality [13,29] as well as models that link rapid feedforward processing of spike timings with phase-locked gamma oscillations [30]. Furthermore, GBRs seem to be facilitated when participants are required to make a behavioral response compared to when no responses are required [31]. It might be argued that the observed effects are, although triggered by the stimulus, mainly a manifestation of anticipation of the stimulus. Such an effect should take place already before the stimulus would be applied. It has been reported that such anticipation effects vary with the randomization procedure used for inter stimulus intervals [19]. Although different randomization procedures were used for the different blocks of the experiment, no effect of the randomization procedure was observed. This favors the interpretation that the electrophysiological effects are related to facilitation of stimulus processing rather than anticipation of stimulus timing in fast response trials.

Due to its similar frequency characteristics, electromyographic activity is usually a big problem when dealing with EEG gamma activity. We visually inspected every trial before analysis to make sure that there was no excessive high frequency activity in the data. Still there might be muscular activity in the data, with an amplitude that is too low to be detected visually. However, the gamma activity in the present study displays one important property that cannot be expected for such low amplitude muscular activity: it is phase-locked to a visual stimulus while showing virtually no power increase. Furthermore, low amplitude muscular activity would be expected to have a constant tonus which would be subtracted with the baseline. Therefore, we believe that the results described here can be related to cerebral processing rather than muscular artifacts.

How do the slow negative potential and the findings about phase-locked gamma oscillations fit together? While slow negative potentials like CNV or readiness potential are usually associated with anticipatory motor activity (for review see [26]), eGBRs have been linked to early visual processing [6]. These two phenomena might be related in two different ways: First, the slow negativity might be a prerequisite for an enhanced eGBR, which in turn enables the participant to perform a rapid response. Second, the slow negativity as well as the eGBR might independently facilitate rapid reactions. The fact, that we did not find significant differences in eGBR for different magnitudes of the slow negativity in single trials, is in line with the second alternative. However, further research in this direction is needed to reveal the exact relations between these two brain signals and behavioral performance. In such an experiment the stimulus preceding ERP and poststimulus gamma band activity could be dissociated by experimentally suppressing one of these phenomena independently by an appropriate experimental manipulation.

We observed very fast reaction times in the current study. Compared to the reaction times of 400–600 ms (e.g. [6,32]) observed in simple cognitive experiments, 200 ms might seem very short. It should be kept in mind, that in the current experiment no stimulus discrimination was necessary. Taking into account the high contrast and very low spatial frequency (one big square, no texture) of the stimuli used in the present study, reaction times around 200 ms fit well with the expected reaction times as estimated by Plainis and Murray [33].

Previous studies reported correlations of either gamma peak latency [12] or amplitude [13] with reaction time. However, in both studies effects were observed after the participant's average response time. Furthermore, these studies did not analyze phase-locking of the activity. Although our data also include late, stimulus induced amplitude modulations, which also vary with reaction time, we show that fast and slow response trials differ with respect to their phase-locking even before amplitude modulations start to play a role. Furthermore, the fact that the AM effect on gamma oscillations only becomes significant after the participants already have responded, excludes the latter from being a causal factor determining the reaction time differences. These results might indicate that phase-locked GBRs play an important role in fast detection of visual stimuli, whereas induced GBRs might be linked to further refinement of this initial classification, as has been suggested by recent models of visual processing [30,34].

The current results demonstrate that rapid visual processing in preparation for a speeded response is (at least partly) dependent on stimulus locked activity in the gamma range. However, the actual behavior in a cognitive task is most probably based on the interactions of different oscillatory processes. Indeed, movement seems to be related to other, probably lower frequencies [35]. Also the integration of different modalities to a coherent movement has been associated with oscillatory activity at lower frequencies [36]. In contrast, the observed effects in the gamma range seem primarily related to visual processing, albeit preparing the brain for speeded responses. It has been argued recently that high frequency oscillations which are evoked in early sensory areas need to be down modulated to lower frequencies that then cover more distributed areas of the brain [37,38]. Thus, it seems plausible to assume that early evoked gamma activity might be necessary but not sufficient for speeded responses and later activity of lower frequency must mediate the results of the enhanced visual processing to motor areas. Indeed, lower frequencies during a visual motor integration task have been reported to be highly synchronized between visual and motor areas in cats [39].

## Conclusion

In conclusion we could show that fast reaction times are associated with enhanced phase-locking in the gamma range. Evoked gamma activity might thus be related to a fast mode of visual processing.

## Methods

### Participants

Thirteen healthy volunteers aged between 22 and 44 years (mean age 27 ± 6.7, 6 m, 7 f) participated in the current study. All participants had normal or corrected to normal vision and were free of current or past neurological or psychiatric disorders. Before the recording session started, participants gave their informed consent to participate. The experimental procedure was in accordance with the guidelines of the local ethics commitee of the university of Magdeburg and the declaration of Helsinki.

### Stimuli and experimental procedure

The participants viewed large black squares (16 × 16 cm at a distance of 120 cm, subtending 8 degree visual arc) that were presented in front of a white background on a TFT monitor (width = 34.5 cm, height = 25.9 cm). Stimuli were presented for 1000 ms. The participants were instructed to press a button as fast as possible, as soon as a square appeared on the screen. After every button press, the participants received feedback about their reaction time. Stimuli were presented in three blocks of 200 trials each. Inter stimulus intervals (ISIs, time interval between offset of one stimulus and onset of the next stimulus) were taken from a uniform random distribution in one block, from a normal distribution in another block and from a shifted exponential distribution in the third block, to control anticipatory effects due to the randomization of the ISI. It has been shown, that stimulus preceding negative potentials are weakest for a uniform distribution of ISIs [19]. In all blocks mean ISI was 1200 ms and standard deviation was 300 ms. Block sequence and response hands were counterbalanced across participants. Participants were instructed to fixate a small black cross in the center of the screen during the whole experiment.

### Data acquisition

During data recording, participants sat in an electrically shielded and sound attenuated room (IAC, Niederkrüchten, Germany). The stimulation monitor was placed outside the recording cabin behind an electrically shielded window. All devices inside the cabin were battery operated to avoid line frequency interference (50 Hz in Germany). EEG activity was measured form 31 scalp locations referenced to the nose. Electrode positions were selected according to an extended 10–20 system. Electrooculographic activity was recorded from an electrode placed below the orbital rim in order to detect eye movement artifacts. Activity was recorded using sintered Ag/AgCl electrodes mounted in an elastic cap (Easycap, Falk Minow Services, Munich) and amplified by means of a BrainAmp amplifier (Brain Products, Munich). Electrode impedances were kept below 5 kΩ. The data was analog low pass filtered at 200 Hz, digitized at a rate of 500 Hz and stored on a computer hard disc for offline analysis. Digitized EEG data was transferred to a computer outside the recording cabin with a fiber optic cable. No analog or digital high pass filter was applied to preserve DC components of the signal. An automatic artifact rejection was computed which excluded trials from further analysis if the standard deviation within a moving 200 ms time window exceeded 40 *μ*V in any channel (EEProbe, ANT, Enschede). The automatic artifact rejection was supplemented by visual inspection to ensure that only trials without artifacts due to eye movements, motor activity or amplifier noise were included in the subsequent analysis.

### Data analysis

For each single trial the response time was recorded. Based on inspection of the response time histograms, only responses between 100 and 400 ms were considered for further analysis. See top of Figure 3 for a histogram of response times across all participants. To investigate the current hypotheses it might sound obvious to perform a correlational analysis. One would expect, that there is a high correlation between the reaction time and the eGBR (and probably the slow negative potential) across trials. The main problem with this approach lies in the definition of evoked activity. Evoked activity is defined as being phase-locked to the onset of the stimulus [14]. Unfortunately phase-locking to the onset of a stimulus cannot be analyzed in single trials. Thus, a correlational analysis of the observed effects could only indirectly be performed on the basis of subaverages. To this end, responses of each participant were split into two groups according to whether the response was faster than the participant's median response time or slower. As neither EEG nor response time data differed significantly between blocks, data from all three blocks were merged for the analysis of EEG. Event-related potentials (ERPs) were computed as averages across trials for fast response and slow response trials separately. The activity in a baseline 500 to 700 ms before stimulus onset was subtracted from the ERPs. To investigate the impact of slow ERP components preceding the stimulus on eGBRs, a straight line was fit to the last 500 ms before stimulus onset in single trials. Based on the slope of this line a median split was performed to obtain trials with strong stimulus preceding negativity and weak stimulus preceding negativity.

To analyze event-related gamma oscillations, a wavelet transform was applied. The wavelet transform was computed at linearly spaced time and frequency positions using a discrete version of the integral wavelet transform with the morlet wavelet (i.e. a modulated gaussian) as basis function. At 40 Hz this wavelet had a time frequency resolution of 2*σ*_*t *_≈ 50 ms and 2*σ*_*f *_≈ 13 Hz. The exact time frequency localization depends on the analyzed frequency. The wavelet transform represents a signal as a function of time and frequency. From these time-frequency representations three characteristic values were derived: (i) the strength of the evoked activity as the time-frequency representation of the ERP signal, (ii) the amount of amplitude modulation (AM, i.e. the total amplitude of the single trials irrespective of the phase^1^) as the average of the amplitudes of the wavelet transformed single trials (iii) the strength of phase-locking (PL). (see Notes)

To quantify PL, the complex wavelet transformed data from each single trial were divided by their amplitude and subsequently averaged. From these complex averages, the modulus was taken as a measure of PL [20,21]. This measure of PL is bounded between 0 and 1, where 1 indicates perfect phase alignment across trials and 0 indicates a constellation in which the phases exactly cancel out each other, as it is the case for a uniform distribution of phases across trials. On average 130 trials were included in the analysis of ERP and oscillatory activity for fast and slow response subaverages. For a uniform distribution of phases across trials the 95th percentile of the phase-locking value was numerically estimated to be ≈0.15. The three measures are described in more detail elsewere [40,41]. From the time frequency representations of eGBR and AM, the average activity from the last 200 ms before stimulus onset was subtracted, to obtain a measure of the event related changes of these quantities. One participant was excluded from the GBR analysis due to large muscular artifacts that could not be separated from the GBR.

For every participant the eGBR was defined as the peak response in a frequency range between 30 and 90 Hz and a time range between 50 and 160 ms after stimulation onset. The frequency range for the eGBR was predefined to include the whole gamma frequency range. The time range was adapted to include all initial phase-locked responses (see Table 1). The analysis was focused on peak responses, because the response frequency in the gamma range has been shown do vary considerably across participants [6]. Responses were pooled into two regions of interest (ROI) as summarized in Table 2. The posterior ROI was chosen to include channels over visual areas, the central ROI was chosen to include channels from a broad area around the central sulcus. We decided to select electrodes from both hemispheres into the ROI, because we observed in a pre-analysis, that no significant laterality effects were present in the data (slow negative potential: *t*(12) = .29, central eGBR: *t*(9) = -0.84, contra- vs. ipsilateral to responding hand). Response strengths and latencies were analyzed by means of ANOVA for repeated measurements with two factors (ROI × SPEED). If for a particular participant and condition no response peak could be extracted, this value was considered "missing" in the statistical analysis. The statistical analysis was performed separately for eGBR, AM and PL.

## Authors' contributions

IF, UK and CSH have planned the experiment. IF and JS recorded the data. IF, JS and NAB analyzed the data. IF, NAB, UK and CSH wrote the manuscript. All authors read and approved the manuscript

## Note

^1^Note that some authors use the term induced activity for this phenomenon. However, induced activity is usually charaterized by not being phase-locked. The measure of AM contains both, phase-locked and non phase-locked signal components.

## Figures and Tables

**Table 1:** Response frequencies and latencies for different participants If a participant did not show any peak at all, this value is marked as "no response" in the table and the value was excluded from the statistical analysis

participant	early (60–140 ms)		late (180–400 ms)	
	frequency [Hz]	latency [ms]	frequency [Hz]	latency [ms]

participant 1	33	140	53	336
participant 2	31	120	no response	
participant 3	33	99	45	328
participant 4	37	69	52	268
participant 5	35	137	42	331
participant 6	38	96	40	294
participant 7	39	90	48	311
participant 8	30	97	40	304
participant 9	33	113	49	279
participant 10	36	72	53	255
participant 11	42	130	44	218
participant 12	61	79	44	300
participant 13	excluded due to high frequency artifacts			

**Table 2:** Regions of interest

ROI	channels
central	TP9, TP10, T7, T8, CP5, CP6, C3, C4, Cz, FC1, FC2, FC5, FC6
posterior	CP1, CP2, P3, P4, Pz, P7, P8, O1, O2
